# Fully Closed-Loop Insulin Delivery in Patients Undergoing Pancreatic Surgery

**DOI:** 10.1089/dia.2022.0400

**Published:** 2023-02-28

**Authors:** Gabija Krutkyte, Jonathan Roos, Daniel Schuerch, Cecilia Czerlau, Malgorzata E. Wilinska, Patrick Y. Wuethrich, David Herzig, Roman Hovorka, Andreas P. Vogt, Beat Gloor, Lia Bally

**Affiliations:** ^1^ Department of Diabetes, Endocrinology, Nutritional Medicine and Metabolism, Bern University Hospital, University of Bern, Bern, Switzerland.; ^2^ Department of Anaesthesiology and Pain Medicine, Bern University Hospital, University of Bern, Bern, Switzerland.; ^3^ Department of Nephrology and Hypertension Inselspital, Bern University Hospital, University of Bern, Bern, Switzerland.; ^4^ Wellcome Trust-MRC Institute of Metabolic Science, University of Cambridge, Cambridge, United Kingdom.; ^5^ Department of Visceral Surgery and Medicine, Bern University Hospital, University of Bern, Bern, Switzerland.

**Keywords:** Closed-loop glucose control, Diabetes technology, Pancreatic surgery, Perioperative glycemic control

## Abstract

The central role of pancreas in glucose regulation imposes high demands on perioperative glucose management in patients undergoing pancreatic surgery. In a post hoc subgroup analysis of a randomized controlled trial, we evaluated the perioperative use of subcutaneous (SC) fully closed-loop (FCL; CamAPS HX) versus usual care (UC) insulin therapy in patients undergoing partial or total pancreatic resection. Glucose control was compared using continuous glucose monitoring (CGM) metrics (% time with CGM values between 5.6 and 10.0 mmol/L and more). Over the time of hospitalization, FCL resulted in better glucose control than UC with more time spent in the target range 5.6–10.0 mmol/L (mean [standard deviation] % time in target 77.7% ± 4.6% and 41.1% ± 19.5% in FCL vs. UC subjects, respectively; mean difference 36.6% [95% confidence interval 18.5–54.8]), without increasing the risk of hypoglycemia. Findings suggest that an adaptive SC FCL approach effectively accommodated the highly variable insulin needs in patients undergoing pancreatic surgery. Clinical trials registration: ClinicalTrials.gov, NCT04361799.

## Introduction

Pancreas surgery remains technically challenging and is associated with considerable morbidity and mortality for all indications.^1^ Perioperative hyperglycemia and glycemic variability, in particular in the early postoperative period, were shown to correlate with postoperative complications.^2^ Owing to the central role of the pancreas in the regulation of blood glucose homeostasis, pancreatic surgery imposes high demands on perioperative glucose management.^3^

Resection of pancreatic tissue not only leads to lower insulin production but also reduces glucagon secretion with total pancreatectomy translating into an absolute deficiency of both insulin and glucagon. As a result, operated individuals are predisposed to marked fluctuations between clinically significant hypo- and hyperglycemia. Glycemic instability in the perioperative setting is further compounded by the frequent use of glucocorticoids, nutrition support, and high workload of hospital staff.^4,5^

We have recently reported that a fully closed-loop (FCL) insulin delivery system, which autonomously adjusts subcutaneous (SC) insulin based on continuously monitored glucose levels, significantly improves perioperative glucose control without increasing the risk of hypoglycemia in patients undergoing various types of elective surgery.^6^ Here we report the results of a subgroup analysis focusing on the efficacy of the SC FCL approach versus usual care (UC) in the complex subgroup of patients undergoing total or partial pancreatectomy.

## Research Design and Methods

### Design

This post hoc subgroup analysis of a single-center open-label randomized controlled trial included 13 participants undergoing total or partial pancreatic surgery at the University Hospital Bern (6 in the FCL group, 7 in the UC group). Details can be found in the original publication.^6^ Participants met the key inclusion criteria comprising (1) expected perioperative insulin requirements, (2) planned surgery duration ≥2 h, and (3) expected length of stay ≥72 h.

All participants provided written informed consent and the protocol was approved by the Ethics Committee Bern, Switzerland (2020-01024). The trial was done in accordance with the principles of the Declaration of Helsinki. The data sets analyzed during this study are not publicly available, but are available from the corresponding author on reasonable request.

### Procedures

The SC FCL system consisted of an Android smartphone, running the Cambridge adaptive model predictive control algorithm (version 0.3.71; HX variant) on the CamAPS HX mobile application (CamDiab Ltd., Cambridge, UK), an SC real-time continuous glucose monitoring (CGM) sensor (Dexcom G6; Dexcom, San Diego, CA), and the SC insulin pump (Dana Diabecare RS, Sooil, South Korea). The CamAPS HX app received CGM glucose values and communicated through Bluetooth with the pump, which modulated the SC delivery of faster acting insulin aspart (Fiasp; Novo Nordisk, Bagsværd, Denmark) every 8–12 min. The FCL insulin therapy was initialized upon hospital admission using the participants' body weight and estimated total daily insulin dose.

The nominal glucose target was set to the default of 5.8 or 7.0 mmol/L, based on individual circumstances. Participants were treated with FCL insulin therapy until hospital discharge or a maximum of 20 days. The glucose levels (recorded through a masked Dexcom G6 CGM) of the UC group were managed by the clinical team in accordance with local guidelines. The study did not interfere or specify the perioperative care plans and activities. All study devices were managed by the study team.

### Outcomes

Outcomes of this subgroup analysis were the level of glucose control (% time spent with CGM values in predefined ranges and mean glucose concentration) and glucose variability (defined as standard deviation [SD] and coefficient of variation of sensor glucose). We further assessed insulin doses in the UC group and investigated the evolution of the insulin requirements in the FCL group after pancreatic resection. Glucose control was evaluated from the time of hospital admission until discharge (or a maximum of 20 days). Results are reported for the overall period as well as separately for the time from admission to end of anesthesia (immediate perioperative period) and from end of anesthesia to discharge (postoperative period).

### Statistical analysis

Aggregated period-specific summary measures (overall, perioperative, and postoperative periods) were calculated and are presented by treatment. Outcomes were compared between treatments using unpaired Welch's *t*-test for variables conforming to normality assumptions, while the Mann–Whitney *U* test was used otherwise. Data are reported as mean difference and 95% confidence intervals (CIs) between the interventions (or median of the differences corresponding to the Hodges–Lehmann estimate and its 95% CI in case of nonparametric testing). Owing to the retrospective nature of the analysis and the unadjusted multiple testing, definite inference cannot be made. As such, no p-values are reported in the main article and results should be considered descriptive/exploratory.

## Results

Characteristics of the patient population including the underlying pancreatic disease, diabetes history, and comorbidity are reported in Supplementary Appendix Table SA1. Participants of both groups were comparable with respect to age, BMI, and American College of Surgeons surgical risk assessment.^7^ Details of perioperative care (including type and duration of surgery), complications, and length of hospital stay are provided in Supplementary Appendix Table SA2. The median time from treatment initialization until end of surgery was 7.0 h [6.1–13.1 h] in FCL and 6.5 h [5.7–9.5 h] in UC groups, respectively. Postoperative period lasted for a median of 16.3 days [12.5–19.2 days] in the FCL and 11.5 days [9.5–15.3 days] in the UC group.

**FIG. 1.**
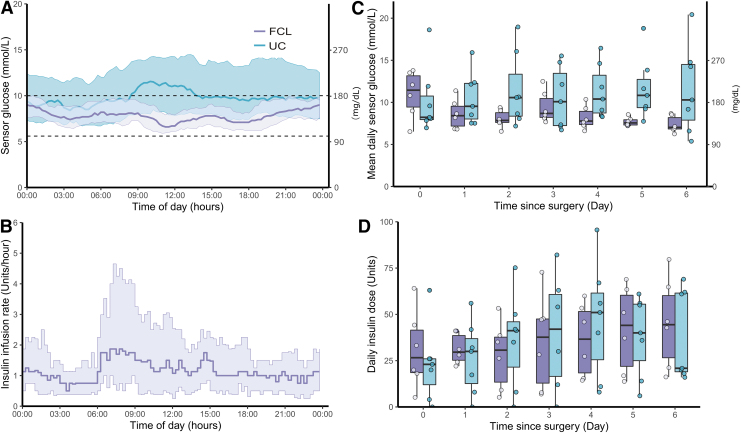
Sensor glucose metrics and insulin doses in the FCL and UC group. **(A)** Ambulatory glucose profile in the FCL (purple) and UC (green) group during the entire study period (from the time of hospital admission until discharge (or a maximum of 20 days), (lines indicate median, shaded areas indicate IQRs). The glucose target range (dashed line) is 5.6–10.0 mmol/L (100–180 mg/dL). **(B)** Twenty-four-hour insulin delivery profile in the FCL group during the entire study period (from the time of hospital admission until discharge (or a maximum of 20 days), (line indicates median, shaded area indicates IQR). **(C)** Evolution of the mean daily sensor glucose in the FCL (purple) and UC (green) group after surgery. **(D)** Evolution of total daily insulin doses in the FCL (purple) and UC (green) group after surgery. Box-plots show individual means (symbols), median values of the group (solid line), IQR (box outline), and spread of data points without outliers (whiskers). Data after day 6 are not shown due to the low number of subjects after this time. FCL, fully closed-loop; IQR, interquartile range; UC, usual care.

Figure 1 illustrates the ambulatory glucose profile (Fig. 1A) as well as insulin infusion rate (Fig. 1B) in the FCL group over the 24 h. The proportion of time with sensor glucose values in the glycemic target range between 5.6 and 10.0 mmol/L throughout the hospital stay was higher in the FCL versus UC group (77.7% ± 4.6% vs. 41.1% ± 19.5%, 95% CI_difference_ [18.5–54.8]). FCL also resulted in lower mean glucose levels (8.2 ± 0.4 mmol/L vs. 11.0 ± 2.9 mmol/L, 95% CI_difference_ [−5.5 to −0.1], see Fig. 1C) and glucose variability (SD 2.2 ± 0.3 mmol/L vs. 3.3 ± 0.7 mmol/L, 95% CI_difference_ [−1.8 to −0.5]) when compared with UC.

The improvement of glucose control was mainly achieved by a reduction of hyperglycemia in the FCL versus UC group (proportion of time with sensor glucose level >10 mmol/L accounted for 15.8% and 49.5% in the FCL and UC groups, respectively) as the time with CGM < 3.9 and < 3.0 mmol/L was low and similar in either group. The average total daily insulin over the entire study duration did not significantly differ between groups with mean doses of 31.4 ± 12.2 U and 34.2 ± 16.7 U in FCL and UC, respectively (95% CI_difference_ [−20.5 to 15.0]).

In the UC group, 85.7% were treated with IV insulin postoperatively for a median duration of 2 days [1.5–4.5 days]. Although no differences in glucose and insulin outcomes were noted in the immediate perioperative period, obvious benefits of the FCL approach in terms of an increase in the time spent with sensor levels between 5.6 and 10.0 mmol/L without increasing the risk of hypoglycemia were observed in the postoperative period (details are provided in Table 1). In the FCL group, mean daily sensor glucose improved over time, whereas no such evolution was seen in the UC group. The postoperative evolution of total daily insulin doses is illustrated in Figure 1D and shows an increase over time in both groups.

**Table 1.** Measures of Perioperative Glucose Control According to Period

	FCL	UC	95% CI for the between-group difference (FCL, UC)
Overall			
Proportion of time with sensor glucose level in the target range [5.6–10.0 mmol/L] (%)	77.7 ± 4.6	41.1 ± 19.5	18.5 to 54.8
<3.0 mmol/L (%)	0.0 [0.0 to 0.0]	0.0 [0.0 to 0.1]	−0.3 to 0.0
<3.9 mmol/L (%)	0.2 [0.0 to 0.3]	0.1 [0.0 to 1.0]	−1.4 to 0.3
<5.6 mmol/L (%)	5.8 [3.6 to 8.3]	4.0 [2.9 to 8.2]	−8.7 to 5.5
>10.0 mmol/L (%)	15.8 [14.5 to 18.2]	49.5 [36.8 to 65.8]	−67.1 to −6.6
>20.0 mmol/L (%)	0.0 [0.0 to 0.0]	1.2 [0.1 to 3.9]	−6.5 to 0.0
Mean sensor glucose levels (mmol/L)	8.2 ± 0.4	11.0 ± 2.9	−5.5 to −0.1
SD sensor glucose levels (mmol/L)	2.2 ± 0.3	3.3 ± 0.7	−1.8 to −0.5
CV sensor glucose levels (%)	26.3 ± 2.2	30.9 ± 5.7	−9.9 to 0.9
Intrasurgery^a^			
Proportion of time with sensor glucose level in the target range [5.6–10.0 mmol/L] (%)	64.1 ± 30.2	58.8 ± 30.5	−32.0 to 42.6
<3.0 mmol/L (%)	0 [0 to 0]	0 [0 to 0]	0.0 to 0.0
<3.9 mmol/L (%)	0 [0 to 1.7]	0 [0 to 0]	0.0 to 2.3
<5.6 mmol/L (%)	0 [0 to 9.6]	0 [0 to 0.0]	0.0 to 12.8
>10.0 mmol/L (%)	20.9 [1.6 to 48.4]	34.2 [17.7 to 49.9]	−54.2 to 39.5
>20.0 mmol/L (%)	0 [0 to 0]	0 [0 to 0]	NA^b^
Mean sensor glucose levels (mmol/L)	8.3 ± 1.7	9.9 ± 2.4	−4.2 to 0.9
SD sensor glucose levels (mmol/L)	1.5 ± 0.6	1.7 ± 0.7	−1.0 to 0.6
CV sensor glucose levels (%)	18.4 ± 8.9	17.8 ± 8.1	−9.9 to 11.2
Postsurgery^c^			
Proportion of time with sensor glucose level in the target range [5.6–10.0 mmol/L] (%)	79.6 ± 6.0	40.0 ± 20.4	20.5 to 58.6
<3.0 mmol/L (%)	0 [0 to 0]	0 [0 to 16]	−0.3 to 0.0
<3.9 mmol/L (%)	0.09 [0.04 to 0.20]	0.15 [0.01 to 1.03]	−1.4 to 0.2
<5.6 mmol/L (%)	5.9 [3.4 to 8.6]	4.0 [1.2 to 8.4]	−8.6 to 6.8
>10.0 mmol/L (%)	14.5 [10.5 to 15.8]	51.5 [36.4 to 68.4]	−74.4 to −6.7
>20.0 mmol/L (%)	0 [0 to 0]	0.98 [0.08 to 4.03]	−7.1 to 0.0
Mean sensor glucose levels (mmol/L)	8.1 ± 0.3	11.1 ± 3.1	−5.8 to −0.1
SD sensor glucose levels (mmol/L)	2.0 ± 0.4	3.2 ± 0.6	−1.9 to −0.6
CV sensor glucose levels (%)	24.0 ± 4.2	30.0 ± 6.5	−12.7 to 0.6

Data are mean ± SD or median [25^th^ to 75^th^ percentile].

^a^ Intrasurgery period duration (h): FCL 7.0 [6.1–13.1]; UC 6.5 [5.7–9.5].

^b^ All participants had 0% time >20 mmol/L, consequently no CI can be calculated.

^c^ Postsurgery period duration (days): FCL 16.3 [12.5–19.2]; UC 11.5 [9.5–15.3].

CI, confidence interval; CV, coefficient of variation; FCL, fully closed loop; SD, standard deviation; UC, usual care.

## Discussion

In this exploratory post hoc analysis, we compared the perioperative glycemic efficacy and insulin requirements of SC FCL insulin delivery with UC insulin therapy in patients undergoing pancreatic surgery. We observed that FCL substantially improved glycemic control by increasing time spent in the glycemic target range and lowering mean sensor glucose without increasing the risk of hypoglycemia.

Although we have recently shown the feasibility and superior performance of the FCL approach over UC insulin regimes in various types of elective surgery, the present analysis provides evidence that an FCL insulin-only approach effectively controls glucose levels in clinical situations with maximum glycemic lability as is the case after total pancreatectomy (five out of six patients in the FCL group received total pancreatectomy in this study). The absolute insulin deficiency that is also the pathophysiological hallmark of type 1 diabetes makes these two populations comparable in terms of complexity of their glucose control.

However, the concomitant lack of glucagon secretion is mainly pertinent to patients with total pancreatectomy, thereby further challenging glucose management in this population. In this context, the present results are encouraging as previous efforts to control glucose levels by an FCL approach in people with type 1 diabetes have shown insufficient performance^8–10^ due to the relatively slow absorption and action of insulin after SC insulin delivery.^11^

However, it is important to note that the algorithm used in this study has a considerably enhanced adaptivity to allow for more responsive insulin dosing compared with currently available hybrid closed-loop systems for the treatment of type 1 diabetes. In addition, the amount of oral dietary intake in the perioperative setting is likely lower than in usual outpatient conditions.

We are not aware of any other study that analyzed the use of an SC FCL insulin-only system to manage perioperative glucose levels in patients undergoing pancreatic surgery. Nevertheless, observational studies and a nonrandomized clinical trial investigated the efficacy of STG-55 (Nikkiso, Inc.) FCL that uses the intravenous route for both glucose sensing and insulin delivery.^12,13^ The complexity of its operation and high blood requirements for sampling (2 mL/min) restrict its application to very short postoperative stays in intensive care units (e.g., 48 h).

Although these small studies reported benefits in terms of reducing postoperative complications in pancreatic surgery patients, evidence from randomized controlled trials is still lacking. The advantages in terms of facilitation of care by FCL systems, however, are more apparent. Pancreatic resections are high-risk procedures with frequently experienced complications (30%–73%) and high complexity of postoperative care.^14,15^ This is reflected by long length of hospital stays that encompassed 15 days in this study, in line with recently published literature.^1^ Complex patient needs further translate into a high workload burden of hospital staff that may worsen during times of resource shortages (e.g., as experienced during the COVID-19 pandemic).

FCL insulin therapy may, therefore, not only contribute to improved quality and safety of blood glucose control in this challenging population, but also reduce the work load of hospital staff. Of note, a recently published study reported increased time in euglycemia and reduced time in hypoglycemia using a bihormonal SC FCL system modulating both insulin and glucagon compared with UC in 12 outpatients after total pancreatectomy.^16^ Future head-to-head comparative studies of insulin-only versus combined insulin/glucagon SC FCL systems will unravel whether the increased complexity of a dual hormone approach is justified in pancreatectomized patients.

We acknowledge the limitations that are inherent to any post hoc analysis and the fact that the FCL insulin treatment was managed by the study team rather than the hospital staff. In addition, the small sample size needs to be considered. Still, we believe that these early findings provide an important steppingstone for future larger and well-designed studies in the field of perioperative and possibly postoperative glucose management in patients undergoing pancreatic surgery.

## Supplementary Material

Supplemental data

Supplemental data

## Supplementary Material

Supplementary Appendix Table SA1

Supplementary Appendix Table SA2
