# Supplementary material for: Fully Closed-Loop Insulin Delivery in Patients Undergoing Pancreatic Surgery
Source: Diabetes Technol Ther. 2023 Feb 28;25(3):206–11. doi: 10.1089/dia.2022.0400 (PMC9983122; doi:10.1089/dia.2022.0400)
Supplement: Supplemental data [file Suppl_AppendixSA1.docx]

**Supplementary Appendix**

| **Table S1. Baseline characteristics.** |  |  | |
| --- | --- | --- | --- |
|  | **FCL (n=6)** | **UC (n=7)** | |
| n | 6 | 7 | |
| Age (years) | 67±25.4 | 72.4±8 | |
| Sex (female participants) | 4 (67) | 3 (43) | |
| BMI (kg/m^2^) | 27.4±5.7 | 27.9±3 | |
| HbA1c (%) | 8.2±2 | 7.4±2 | |
| HbA1c (mmol/mol) | 59.4±16.1 | 57.7±22.4 | |
| Diabetes duration (years) | 6.5±12.9 | 5.2±7.2 | |
| Duration of insulin therapy (years)^a^ | 0.1±0.1 | 1.9±3 | |
| *Glucose lowering therapy at enrolment* |  |  | |
| Basal insulin therapy | 2 (33.3) | 1 (14.3) |  |
| Basal-bolus insulin therapy | 2 (33.3) | 3 (42.9) |  |
| Insulin-naive | 2 (33.3) | 3 (42.9) |  |
| Metformin | 1 (16.7) | 3 (42.9) |  |
| Gliptin | 1 (16.7) | 2 (28.6) |  |
| GLP-1 RA | 0 (0) | 0 (0) |  |
| SGLT-inhibitor | 1 (16.7) | 1 (14.3) |  |
| Sulfonylurea | 1 (16.7) | 0 (0) |  |
| Charlson Comorbidity Index | 6.2±3.2 | 7.3±2.3 | |
| ACS risk of complications (%) | 31.9±8 | 31±7.4 | |
| *Underlying pancreatic disease* |  |  | |
| Pancreatic ductal adenocarcinoma | 5 (83.3) | 4 (57.1) | |
| Intraductal papillary mucinous neoplasm | 0 (0) | 3 (42.9) | |
| Hereditary chronic pancreatitis | 1 (16.7) | 0 (0) | |
| Data are mean±SD or n (%) unless otherwise specified. ACS, American College of Surgeons; BMI, Body-mass index; GLP-1 RA, Glucagon-like Peptide 1 receptor-agonist; SGLT, Sodium-glucose co-transporter.  ^a^Insulin-naive patients are not considered. | | | |
