# Supplementary material for: Fully Closed-Loop Insulin Delivery in Patients Undergoing Pancreatic Surgery
Source: Diabetes Technol Ther. 2023 Feb 28;25(3):206–11. doi: 10.1089/dia.2022.0400 (PMC9983122; doi:10.1089/dia.2022.0400)
Supplement: Supplemental data [file Suppl_AppendixSA2.docx]

| **Table S2. Characteristics of perioperative care.** |  |  |
| --- | --- | --- |
|  | **FCL (n=6)** | **UC (n=7)** |
| *Type of Surgery* |  |  |
| Laparascopic Duodenopancreatectomy | 1 (16.7) | 2 (28.6) |
| Laparascopic Total Pancreatectomy | 0 | 1 (14.3) |
| Open Remnant Total Pancreatectomy | 1^a^ (16.7) | 1^b^ (14.3) |
| Open Total Pancreatectomy | 4 (66.7) | 2 (28.6) |
| Open Distal Pancreatectomy | 0 | 1 (14.3) |
| Surgery duration (min) | 383±36 | 353±92 |
| Need for revisional surgery^c^ | 2 (33.3) | 2 (28.6) |
| Pre-surgery carboloading | 5 (83.3) | 0 |
| Intraoperative IV insulin | 1 (16.7) | 7 (100) |
| Postoperative IV insulin | 1 (16.7) ^d^ | 6 (85.7) |
| Duration of postoperative IV insulin (days) | 6^e^ | 2 [1.5; 4.5] |
| Pre-/intraoperative IV glucocorticoids | 5 (83.3) | 7 (100) |
| Postoperative nutritional support | 5 (83.3) | 7 (100) |
| Oral nutritional supplements | 4 (66.7) | 6 (85.7) |
| Enteral tube feeding | 2 (33.3) | 3 (42.9) |
| Parenteral nutrition | 5 (83.3) | 6 (85.7) |
| Planned postoperative IMC stay | 6 (100) | 7(100) |
| IMC stay due to deterioration | 1 (16.7) | 1 (14.3) |
| Length of stay (days) | 17 [13.5; 20.5] | 12 [10; 18.5] |
| *Clavien-Dindo Index ^f^* |  |  |
| Grade 0 | 1 (16.7) | 0 |
| Grade I | 2 (33.3) | 3 (42.9) |
| Grade II | 12 (200) | 14 (200) |
| Grade III | 0 | 2 (28.6) |
| Grade IV | 2 (33.3) | 0 |
| Comprehensive Complication Index | 30.8 [29.9; 46.5] | 29.6 [25.3; 34.5] |
| Data are n (%), mean±SD or median [25^th^; 75^th^ percentile] unless otherwise specified. IV, intravenous, SC, subcutaneous, IMC, Intermediate Care Unit.  ^a^ Open remnant total pancreatectomy after past duodenopancreatectomy performed 5 years ago.  ^b^ Open completion pancreatectomy performed as a revisional surgery due to postoperative anastomotic insufficiency of pancreaticojejunostomy with residual pancreatitis after duodenopancreatectomy during the same hospitalization.  ^c^ Causes for revisional surgeries in both groups: necrosis of the gastric wall; small intestine perforation; R1 resection in postoperative histology specimen after R0 finding in the frozen section; anastomotic insufficiency of pancreaticojejunostomy and acute pancreatitis in the pancreatic remnant  ^d^ Transient IV insulin use due to clinical deterioration with anasarka according to the decision of the clinical team.  ^e^ Duration of postoperative IV insulin therapy reported for one patient during study suspension.  ^f\|^ Note: one subject may be attributed to more than one grade, except for grade 0. | | |
